# The commercialization of university-based research: Balancing risks and benefits

**DOI:** 10.1186/s12910-015-0064-2

**Published:** 2015-10-14

**Authors:** Timothy Caulfield, Ubaka Ogbogu

**Affiliations:** 461 Law Centre, University of Alberta, 111 St and 89 Ave, Edmonton, AB T6G 2H5 Canada; 431 Law Centre, University of Alberta, 111 St and 89 Ave, Edmonton, AB T6G 2H5 Canada

**Keywords:** Commercialization, Pressure to commercialize, Applied research, Knowledge translation, Risks versus benefits, Science hype, Ethics, Science policy, University research, Science funding

## Abstract

**Background:**

The increasing push to commercialize university research has emerged as a significant science policy challenge. While the socio-economic benefits of increased and rapid research commercialization are often emphasized in policy statements and discussions, there is less mention or discussion of potential risks. In this paper, we highlight such potential risks and call for a more balanced assessment of the commercialization ethos and trends.

**Discussion:**

There is growing evidence that the pressure to commercialize is directly or indirectly associated with adverse impacts on the research environment, science hype, premature implementation or translation of research results, loss of public trust in the university research enterprise, research policy conflicts and confusion, and damage to the long-term contributions of university research.

**Summary:**

The growing emphasis on commercialization of university research may be exerting unfounded pressure on researchers and misrepresenting scientific research realities, prospects and outcomes. While more research is needed to verify the potential risks outlined in this paper, policy discussions should, at a minimum, acknowledge them.

## Background

The university research community has always been under various forms of outside pressure – political, economic, and institutional – that has had the potential to impact, for better or worse, the nature and direction of academic research. And, of course, researchers bring their own biases, ambitions and tendencies to their work. Academic research is rarely, if ever, truly pure.

In recent years, however, a new type of pressure has descended on university-based research by way of increased emphasis on the commercialization of research [1–6]. The political mandate that pushes for this shift seems qualitatively different from other pressures. It is not only ubiquitous [1, 4], but it has also been urged, framed and promoted at the highest levels of policymaking [1, 2, 4]. To cite one high profile example, President Obama has enthusiastically endorsed using academic research to drive economic growth in his last two State of the Union Addresses [7, 8] and in other recent speeches [4, 9, 10], and has remarked that “[t]wenty-first century businesses will rely on American science and technology, research and development [7]. Other world leaders have honed in on the message as well [1, 11]; Prime Minister Stephen Harper of Canada has stated, for example, that a primary aim of scientific research is to “power” commerce [12].

Two areas where the push for increased commercialization of university-based research appear particularly evident are research funding and program support. In Canada, for example, there are very few research funding opportunities that are not touched by the commercialization ethos [1]. If researchers want funding, they will now need to frame their work in a manner that accords with the growing political view that universities ought to play a central role in the growth of economies [13, 14], or to accord with national or regional economic priorities [1, 15, 16]. With regard to program support, universities are facing increased pressure to cut programs that do not generate revenue as a way of dealing with budgetary constraints. As recently stated by a Canadian provincial finance minister in a budget presentation speech, universities must “preserve…high value programs and, correspondingly….identify and shed low-value programs that do not represent good return on investment” [17].

Studies of research policy trends suggest that the commercialization ethos and associated pressures are unlikely to relent anytime soon [14] and may, in fact, become the central or defining mission of university-based research. These studies also show that the push to commercialize is almost always presented as an unqualified social good [1] that warrants broad governmental and institutional focus and support [13]. Conversely, the goals and endpoints of the commercialization push are not well articulated, if at all, and its potential risks and challenges are largely absent from policy statements and discussions [1].

This paper highlights the potential risks associated with the commercialization ethos. While many of these risks outlined below require further research to confirm their existence and impacts in connection with the push to commercialize, we are of the view that, at the very least, they should be part of the public and policy dialogue about the role that commercialization should or ought to play in shaping and influencing the direction and focus of university-based research. The risks presented below are by no means exhaustive, or necessarily specific to research commercialization trends. However, they provide critical starting points for a more balanced reflection on the commercialization imperative.

## Discussion

Before engaging with the potential risks of the push to commercialize university research, it is important to acknowledge and highlight some of the benefits and advantages associated with the trend. Commercialization is a primary means through which medical products and services reach the market and consumers, which can, in turn, advance public health. Also, research commercialization initiatives *can*, in theory, generate interest and investment in emerging areas of research, with consequent gains or improvements in research funding, job creation, scope and quality of innovation, creation and growth of industries, and economic sustainability of universities [3, 18, 19]. Commercialization *can* also help build university-industry collaborations that are often necessary for the translation of research into beneficial products and therapies for public use. While a critical or extensive engagement with the positive outcomes of commercialization are beyond the scope of this paper, we acknowledge that there are well established and observed impacts of research commercialization that justify its relevance to research/science policy and society in general. In particular, it is worth noting that our analysis neither touches upon nor seeks to diminish any positive effects associated with the “entrepreneurial university” [20, 21], or the established role of commercialization in advancing university-industry-government relationships (i.e., the “triple helix” concept) [22].

While the afore-mentioned benefits are often upheld in policy documents and discussions as justifications for increased commercialization, they are either expressed in broad aspirational terms or as axiomatic endpoints, and often with little or no evidence orreasoned reflection in support [1, 4, 23]. Moreover, evidence of a link between university research and economic growth is tenuous at best [4, 23, 24], and the discussion of some potential benefits, such as increased industry participation in research translation, fail to acknowledge or discuss established and potential risks, such as risks associated with the data reporting and publication practices in industry funded research.

### Potential risks

#### Adverse impacts on research environment

Two distinct impacts on the research environment arise in connection with commercialization pressure, namely impacts on the direction and type of research being done, and impacts on research practices. Regarding the former, recent studies of the impact of commercialization pressure on the research environment suggest that the trend is viewed unfavourably by many in the research community, and is associated with a variety of adverse effects on the conduct, integrity or direction of research [25–27]. For example, a 2014 Pew Research Center survey of members of American Association for the Advancement of Science, which is the world’s largest general scientific association, found that nearly half (47 %) believed that the pressure to develop marketable products was having an undue influence on the direction of their research, while a majority (69%) viewed a focus on projects expected to yield rapid results as having a similar influence on their research [28]. Another study, from 2014, concluded that the “government-initiated emphasis on commercialization” of U.S. university research “may undermine open paths toward novel technologies and hinder explorations of unknown fields” [29]. A small study of members of the Canadian stem cell research community found similar views and concerns, and that a majority of those surveyed (76 %) either agree or strongly agree that commercialization and translation pressure could impact public trust or funding of their research [1]. In relation to impacts on research practices, several studies have found or posited associations between commercialization activity and data withholding [30–32], the erosion of collaborative research relationships [33, 34] and reluctance or unwillingness to engage in certain research trends, such as open science initiatives, which seemingly conflict with the financial considerations that underlie the pursuit of commercialization [29, 35–37].

#### Science hype

There is growing concern that representations of biomedical research are inappropriately overstated, including in scientific abstracts [38], institutional press releases [39], newspaper coverage of research [40], and in popular culture more broadly. There are, of course, many sources of science “hype” that work in concert to produce exaggerated representations, including publication pressures and public, media and institutional expectations [41]. It is, no doubt, a complex problem involving many actors. But given that the commercialization mandate requires science that can be translated quickly and that will have practical results, it seems likely it is a pressure that is, at least, playing a role in this trend. Some of the concerns associated with science hype include exaggerated public and patient expectations, misinformed science and health policy, and the possibility of loss of public support where promised outcomes do not materialize [42–44]. Science hype can also be exploited by those seeking to market unproven and potentially harmful therapies. Lastly, participation by researchers in promoting or advocating for the commercial potential of their work, especially if tied to success in obtaining research grants, could skew the framing of research questions and outcomes in a manner that compromises their ability to approach the research from a disinterested position.

#### Premature implementation and use of services

A commonly held view among advocates of the push to commercialize is that research activities, particularly in the biomedical and medical sciences, can and ought to yield near-term commercializable products and services for clinical use [1, 2]. While there is a possibility that this position is mere rhetorical flourish in support of arguments for the increasing policy emphasis on translational research, one thing is clear: the view does not accord with any realistic assessment of the process and duration of translational research. Research results do not materialize according to a set schedule or in response to the expectations and demands of the market. Studies, especially of the biomedical and clinical research context, suggest that moving from bench to market takes decades [45, 46], and only few among many research studies yield more than modest direct returns or useful products or services [2, 46, 47]. Even the process of regulatory scrutiny and approval takes years.

Given this reality, the notion that “fast and successful” translation of research results is possible and desirable, especially when expressed by prominent policymakers, may skew research incentives and public expectations in favour of premature implementation of research results, with consequent risks to research integrity and to the quality, safety or efficacy of research outputs [13, 48]. Indeed, commercialization activities and associated incentives have been linked to premature implementation of gene therapy research programs [49]. In the stem cell research context, a global market for putative, scientifically unproven stem cell therapies has emerged, and appears to be expanding in availability and reach despite evidence of considerable health, financial, regulatory and social risks posed by the phenomenon [50–54]. While it remains unclear how the “near-term translation and commercialization” ethos factors into or drives the market for unproven and unapproved stem cell therapies, the phenomenon is arguably a response to the commercialization message. 

Regarding public expectation, the view that near-term translation is possible and achievable, coupled with offerings of premature products and services, may shift public perception of the translational research process or lead to public demand for faster access to research outputs. This may, in turn, create a challenge for regulators both in terms of responding to access demands and justifying regulatory rules and decisions. In the United States, for example, the Food and Drug Administration has recently been forced to use litigation and enforcement orders and notices to deal with premature implementation issues arising in part from consumer demand for stem cell-based products and services [55–58].

#### Public trust

The effect of the commercialization ethos on public trust is less likely to be direct and more consequential to the materialization of some of the afore-discussed risks, such as where overstated research benefits fail to emerge or premature products and services cause harm to the members of the public. However, there is evidence to suggest that commercial considerations negatively affect public perceptions of research, and may have a role to play in the erosion of public support of research and the acceptance of research results [59–62]. In this regard, several academic commentators and research studies have noted or found associations between commercialization activities and declining public trust in research. For example, a recent Australian study of public views of stem cell research concluded that “the results of this research clearly highlight the importance of trust in public support for stem cell research, and that the commercial research context is a significant threat to this trust” [59]. The study also found that “[r]egardless of where scientists source their cells, public support and trust are eroded when their work is conducted in a private research organisation using private funds” [59]. Similarly, a 2012 study of over 1000 Albertans found that, in the context of tissue research, public trust in university research erodes quickly if there are ties to industry [63]. Whether direct or consequential, the potential for loss of public trust is arguably the most pressing risk associated with the drive to commercialize. Given the role that public trust plays in relation to setting research priorities, agenda and in the allocation of research resources, loss of public confidence and trust will most likely doom an affected area of research, or at least create significant setbacks [49, 64]. Indeed, the push toward commercialization seems to be happening at the worst possible time, when public trust in the traditional sources of science is eroding [65]. The further commercialization of university-based research will only exacerbate this dilemma.

#### Skewed health policy

The commercialization agenda clearly favours products and services that can generate economic activity, such as marketable tests, interventions and drugs. This approach arguably has little to do with addressing broader socio-economic and behavioural changes that would have broader and more enduring impact on human health [66]. The potential for harm caused by the push toward commercially oriented technological approaches – at the expense of a focus on social change – has been noted by numerous commentators in the context of a range of emerging technologies – including genetic testing [67], personalized medicine [66], nutrigenomics [68], epigenetics [48] and stem cell research [13]. As noted by Petersen and Krisjansen, “[i]ncreasing investment in the promissory life sciences, including the harnessing of the publicly funded resources of universities and other publicly funded research institutions, means that fewer resources are available for social research and policy initiatives in areas of pressing need” [48]. Given that research on prevention, public health and behaviour intervention already receives far less government funding than research aimed toward treatment [69], such pressure can hardly be viewed as constructive.

Other studies have found that the emphasizing a commercializable, technological approach to health problems may also have an adverse impact on the development of social policy. It has been found, for example, that framing health issues, such as obesity, as being a disease associated with genetics – a framing that is more likely when commercialization is the goal – as opposed to a social problem demanding social reforms, leads to less support for government public health policies [70, 71].

In addition, commercialization may result in clinical trends – such as a push toward more testing and screening procedures – that conflict with other, evidence-based, approaches to healthcare, such as the recent push to do less diagnostic screening in an effort to avoid unnecessary harm, anxiety and costs [72]. And, of course, the push to commercialize may also lead to increase healthcare costs, as the introduction of new technologies almost inevitably do – an ironic conclusion, given that economic benefit is one of the stated benefits of the commercialization agenda.

#### Damage to the long-term economic contributions of university research

The notion that university research should and ought to focus on near-term translational research activities that produce commercializable products and services may obliterate support for other long-term contributions to basic and clinical research that produce both incremental and ground-breaking advances [2, 14]. While this may not in fact be a huge loss if the near-term commercialization strategy is successful (but they rarely, if ever, are), there is little reason to suppose that simply reducing the time for research and development will yield more contributions for the financial or knowledge economies. As one well-known Canadian commentator has noted, for example, the money generated by the intellectual property owned by Canadian universities is minuscule [24]. After accounting for the direct costs associated with the management of commercialization activities, the total surplus for all Canadian universities for 2010 amounted to $2.1 million, and the average income for each university as was only $425,000. This is hardly the kind of money that will drive economies and change the fate of universities. Besides, a focus on near-term commercialization goals may alter research behaviour and incentives and lead to negative impacts on the knowledge economy, such as increased publication and translation of research of diluted value [73, 74] and shifts away from new ideas that require basic research [75].

## Conclusions

There are interesting questions about the degree to which the concerns we note above can be supported by empirical evidence. For example, to what degree is commercialization pressure adding to science hype and a shift in health policy? These are intriguing issues that warrant further research. But regardless, the concerns noted have sufficient empirical backing to suggest that are, at least, worthy of consideration. Indeed, the concerns outlined above have at least as much supporting evidence as the alleged benefits of commercialization and translation pressure. While there has been some acknowledgement and discussion of the risks or trade-offs associated with commercialization, especially in the academic literature, there remains a notable lack of emphasis in the public and policy discussion, at least compared to the emphasis on benefits. The discussion of risks also appears missing in other relevant contexts, such as within university settings. As such, our modest recommendation is that these, and other concerns, be an explicit part of the public, policy and institutional debates surrounding the commercialization of science. There is also a need to highlight and promote established strategies for minimizing commercialization risks, such as increased and better monitoring of university-industry relationships, mechanisms to reduce research hype, increased government funding of independent research and the use of independent grant review mechanisms to test and mitigate commercialization claims.

## Summary

−University researchers are facing increased political and institutional pressure to focus on commercializable research and to rapidly commercialize their research outcomes.
−The push or pressure to commercialize is ubiquitous, and is often presented as an unqualified social good that deserves unique governmental and institutional focus and support.
−There is growing evidence of potential risks flowing from or associated with the push to commercialize, but consideration of such risks are largely absent from policy statements and discussions.
−While more research is needed to confirm the existence of these potential risks, they should, at a minimum, be part of the public and political dialogue about the role of commercialization in shaping the focus and direction of university research.
